# Exchange Coupling Effects on the Magnetotransport Properties of Ni-Nanoparticle-Decorated Graphene

**DOI:** 10.3390/nano13121861

**Published:** 2023-06-15

**Authors:** Erick Arguello Cruz, Pedro Ducos, Zhaoli Gao, Alan T. Charlie Johnson, Dario Niebieskikwiat

**Affiliations:** 1 Departamento de Fisica, Colegio de Ciencias e Ingenierias, Universidad San Francisco de Quito, Quito 170901, Ecuador; earguell@andrew.cmu.edu (E.A.C.); dniebieskikwiat@usfq.edu.ec (D.N.); 2 Department of Physics and Astronomy, University of Pennsylvania, Philadelphia, PA 19104, USA; zlgao@cuhk.edu.hk (Z.G.); cjohnson@physics.upenn.edu (A.T.C.J.)

**Keywords:** graphene, nanoparticles, magnetoresistance, weak localization, exchange coupling

## Abstract

We characterize the effect of ferromagnetic nickel nanoparticles (size ∼6 nm) on the magnetotransport properties of chemical-vapor-deposited (CVD) graphene. The nanoparticles were formed by thermal annealing of a thin Ni film evaporated on top of a graphene ribbon. The magnetoresistance was measured while sweeping the magnetic field at different temperatures, and compared against measurements performed on pristine graphene. Our results show that, in the presence of Ni nanoparticles, the usually observed zero-field peak of resistivity produced by weak localization is widely suppressed (by a factor of ∼3), most likely due to the reduction of the dephasing time as a consequence of the increase in magnetic scattering. On the other hand, the high-field magnetoresistance is amplified by the contribution of a large effective interaction field. The results are discussed in terms of a local exchange coupling, J∼6 meV, between the graphene π electrons and the *3d* magnetic moment of nickel. Interestingly, this magnetic coupling does not affect the intrinsic transport parameters of graphene, such as the mobility and transport scattering rate, which remain the same with and without Ni nanoparticles, indicating that the changes in the magnetotransport properties have a purely magnetic origin.

## 1. Introduction

Graphene has been one of the most studied materials over the last 20 years, due to its linear dispersion relation close to the Fermi level which gives it unique properties. Graphene exhibits a high intrinsic mobility [1,2], can sustain very high current densities [3,4], is a remarkable thermal conductor [5,6], is impermeable to gases [7,8], and can be a benchmark for quantum phenomena such as the room temperature quantum Hall effect [9], the fractional quantum Hall effect [10], and the non-zero Berry phase at low temperatures [11]. Furthermore, developments in the large-scale production of graphene through chemical vapor deposition (CVD) [12] have sparked interest from areas such as medicine [13,14], electronics [15,16,17], optics [18,19], plasmonics [20,21,22,23], and metrology [24].

There has been some research on the magnetic properties of graphene coupled with magnetic materials. A Rashba effect has been observed for CVD graphene grown on nickel [25,26], nickel–gold [27,28], and cobalt [29,30]. This effect suggests a spin–orbit (SO) coupling from a hybridization between the graphene π orbitals and the metal 3d states. Furthermore, ferromagnetic insulators deposited on graphene induce magnetic correlations with an energy on the order of 5 meV [31,32]. This effective exchange field (EEF) has been shown to affect the magnetoresistance [33,34] and Hall resistance [35] in graphene. Control over SO coupling and EEF is important for spintronic devices; therefore, heterostructures of graphene and magnetic materials have been of interest recently.

In this work, we study the effect of ferromagnetic nickel nanoparticles on the magnetoresistance of graphene ribbons under applied magnetic fields of up to 3T and temperatures above 60K. The quantum linear magnetoresistance (QLMR) model of graphene, proposed by Abrikosov [36], is commonly used to describe the interactions between electrons when moving in closed circular orbits due to the effects of the applied magnetic field. However, this effect requires both high magnetic fields (larger than 4T) and low temperatures (below 10K) [37]; thus, the QLMR effect is outside the range of parameters explored in the present work. Therefore, here we analyze the results in terms of a semi-classical model considering the Altshuler–Aronov electron–electron interactions (EEI) and quantum weak localization (WL) phenomena. Our results demonstrate that the magnetotransport properties of graphene are largely affected by the magnetic coupling with the Ni nanoparticles. This coupling is characterized by an exchange constant J∼6meV, which agrees very well with previous measurements and estimations obtained in similar systems [31,32,33,34,35].

## 2. Materials and Methods

### 2.1. Electrode Patterning

The substrate used was a standard 4 inch phosphorus-doped silicon wafer with a 285 nm thick thermal silicon dioxide (SiO_2_) layer. Standard mask photolithography using an SUSS Micro-Tec MA6 Mask Aligner and thermal evaporation using a Kurt J. Lesker PVD 75 e-Beam/Thermal Evaporator were used to define the electrode pattern. First, the wafer was prebaked at 210 °C for 5min to remove any accumulated hydration. A protective layer of polymethylglutarimide (PMGI, Microchem) was spin-coated on the wafer at 4000rpm for 45 s, and then it was baked at 210 °C for 5 min. This avoids contamination from the resist sticking to the graphene surface. Subsequently, an s1813 (Shipley) photoresist was spin-coated on top of the PMGI layer at 5000rpm for 45 s and then it was baked at 100 °C for 5 min. The electrode pattern was defined using photolithography and developed using MF-319 (Microposit). Finally, 5 nm of chrome and 40 nm of gold were thermally deposited, followed by a lift-off process, such that the thin layer of chrome provides higher adhesion of the gold electrodes to the SiO_2_ surface.

### 2.2. Graphene Growth

Graphene was grown on a copper foil substrate (Alfa Aesar, 99.8%, 25 μm thickness) within a low pressure 4 inch chemical vapor deposition tube furnace (OTF1200X-4-C4-SL-UL, MTI). In order to remove any microscopic impurities from the substrate foil, it was cleaned in a 5.4% HNO_3_ bath and sonicated for 45 s. The foil was then washed with two deionized water baths, dried with high pressure N_2_ gas, and loaded into the CVD system. The pressure in the tube furnace was pumped down to 50 mTorr and the temperature was increased at 8 °C/min until reaching an operating temperature of 1020 °C. The copper foil was then annealed at this temperature for 30 min under a flow of 80sccm of H_2_ gas and 500sccm of Ar gas. After the annealing process, CH_4_ was used as a carbon source with a flow of 5sccm for 5 min followed by 10sccm for 10 min, alongside the previous gas flows. The furnace was then rapidly cooled to room temperature under the same flow conditions, thus allowing the graphene to form without reacting back into amorphous carbon. This process has been shown by Johnson et al. to consistently yield large area high quality graphene for multiple types of applications [38,39,40,41,42,43,44,45].

The copper foil with grown graphene was then loaded into a Kurt J. Lesker PVD 75 e-Beam/Thermal Evaporator and a 2 Å thick layer of nickel was deposited on top of the graphene. This last step was skipped for the control sample.

### 2.3. Film Transfer

We used the hydrolysis bubble method to transfer the graphene/nickel (Gr/Ni) film from the copper foil to the Si/SiO_2_ patterned wafer. A sacrificial polymethylmethacrylate (PMMA) support layer of 500 nm thickness was spin-coated on top of the Gr/Ni on copper foil, followed by a baking process for 2min at a temperature of 105 °C. The sample was then connected to the cathode of a voltage power source, and the anode was a platinum wire submerged in a 50 mM NaOH electrolyte solution. The sample was then inserted into the solution as an electric potential difference of 20 V was applied between the terminals. Bubbles that formed at the interface of the copper foil mechanically separated the Gr/Ni/PMMA film from the copper substrate. The film was washed in three sequential DI water baths before transferring it onto the patterned wafer. After air drying and baking at 150 °C for 3min, the sample was submerged in acetone overnight in order to remove the PMMA layer.

### 2.4. Photolithography and Annealing

We fabricated the GR and Ni-GR resistors using standard mask photolithography as described above. A protective layer of PMGI was spin-coated at a spin rate of 4000rpm for 45 s onto the chip and then baked at 125 °C for 5min. Then, an s1813 photoresist was spin-coated at a rate of 5000rpm for 45 s onto the chip and baked at 100 °C for 2min and developed using an MF-319 (Microposit). Excess graphene outside the channel regions was removed via reactive ion etching of oxygen plasma. A Technics RIE-1 was used, filled with O_2_ gas at 1.25Torr, and set at an ionizing power of 50 W for 35 s. Subsequently, the photoresist was removed from the graphene channels using an N-methylpyrrolidinone photoresist remover (NANO Remover PG, MicroChem) and cleaned with acetone and IPA baths.

Finally, the wafer was annealed at 225 °C for 60min under a flow of 250sccm of H_2_ and 1000sccm of Ar, in order to form Ni nanoparticles on the graphene layer through the coalescence of the Ni film. This process resulted in disordered Ni nanoparticles with diameters of around 6 nm.

### 2.5. Sample Characterization

Atomic force microscopy (AFM) images were obtained on an Asylum MFP-3D Origin microscope in AC tapping mode with a 10 nm tip diameter and over scan areas of about 1 μm×1 μm. We used Gwyddion for scanning probe microscopy to process AFM measurements into height profiles and height histograms.

We performed the electrical characterization of the samples in a cryogen-free Versalab system from Quantum Design with applied magnetic fields of up to 3 T (perpendicular to the plane of the graphene sheet) and temperatures between 60K and 140K. The four-point resistance was measured using a Keithley 6221 current source (current 0.1 μA) and an Agilent 34420A nanovoltmeter.

## 3. Theoretical Background

Electrical transport in graphene is commonly described with the semi-classical Drude model; however, corrections must be included in order to explain the occurrence of a field-dependent resistivity. This correction depends on EEI and is considered within the conductivity tensor in two dimensions, and does not contribute to the Hall current [46]. The longitudinal resistivity $\rho_{xx}$ is calculated at the lowest order in the magnetic field *B*, resulting in

(1) $$\begin{array}{cc} \rho_{xx}\left(B\right)= & \rho_{D}+\rho_{D}^{2}\left(\mu^{2}B^{2}-1\right)\Delta \sigma_{\mathrm{EEI}} \end{array}$$

where $\rho_{D}$ is the classical Drude resistivity, μ is the carrier mobility, and the EEI correction to the conductivity, $\Delta \sigma_{\mathrm{EEI}}$, is given by the following equation [47,48,49]

(2) $$\begin{array}{cc} \Delta \sigma_{\mathrm{EEI}}\left(T,\tau \right)= & \frac{e^{2}}{2\pi^{2}\hbar }\ln\left(\frac{k_{B}T\tau }{\hbar }\right)+\\ \; & \;+\frac{e^{2}k_{B}T\tau }{\pi \hbar^{2}}\left[1-\frac{3}{8}f\left(\frac{k_{B}T\tau }{\hbar }\right)\right] \end{array}$$

where τ is the momentum relaxation time and f(x) is a monotonically decreasing function. $\Delta \sigma_{\mathrm{EEI}}$ is temperature dependent [49], but does not depend on the magnetic field as long as $B<\pi k_{B}T/2\mu_{B}$ [50], a condition that is fulfilled in the whole range of temperatures and magnetic fields presented in this work.

On the other hand, WL phenomena are known to be at the root of a distinctive low-field peak in the resistivity observed in graphene [51,52,53,54]. In this effect, two phase-coherent carriers with an opposite wave number move along a closed path and interfere constructively at the initial point, producing backscattering. Thus, the resistivity increases at zero magnetic field [51]. This contribution of WL to the resistivity ($\rho_{WL}$), originating in quantum interference effects [53], is determined by the interplay between the electron scattering and spin-flipping processes. Moreover, the application of a magnetic field disrupts the WL by modifying the electronic quantum phase, thus washing away the possibility for quantum interference such that

(3) $$\begin{array}{c} \frac{1}{\rho_{\mathrm{WL}}\left(B\right)}=\frac{1}{\rho_{\mathrm{WL}}^{0}}+\gamma \;\cdot \;B^{2} \end{array}$$

where both $\rho_{\mathrm{WL}}^{0}$ and γ are determined by quantum interference. Since WL provides an independent scattering mechanism, we consider its contribution to the total resistivity of graphene through the usual Matthiessen rule,

(4) $$\begin{array}{c} \rho \left(B\right)=\rho_{xx}\left(B\right)+\rho_{\mathrm{WL}}\left(B\right) \end{array}$$

In very clean graphene, the process would be slightly different because of the conical Dirac dispersion relation, which introduces an additional Berry phase [55]. Then, in order to observe WL effects, some degree of defects or impurities are necessary, such that the dephasing rate coming from inelastic processes is smaller than the elastic scattering rates of the system. In this way, the phase coherence time would be long enough to allow interference effects to take place. However, an excessive amount of electronic scattering would be detrimental, producing a large Drude resistivity that hides the WL effects (and would also probably end up killing phase coherence). In our case, we found our samples to be in an intermediate situation, where the WL phenomena are clearly observed at small magnetic fields and low temperatures.

## 4. Results

We characterize the magnetoresistance of Ni-nanoparticle-decorated graphene as a function of a perpendicular magnetic field at different temperatures. Given that we are studying the effect of the nickel nanoparticles on the magnetotransport properties of the graphene layer, it is important to verify that during the electrical transport experiments in the Ni-GR sample, the electrical current always runs through the graphene and not through the nanoparticles.

**Figure 1:**
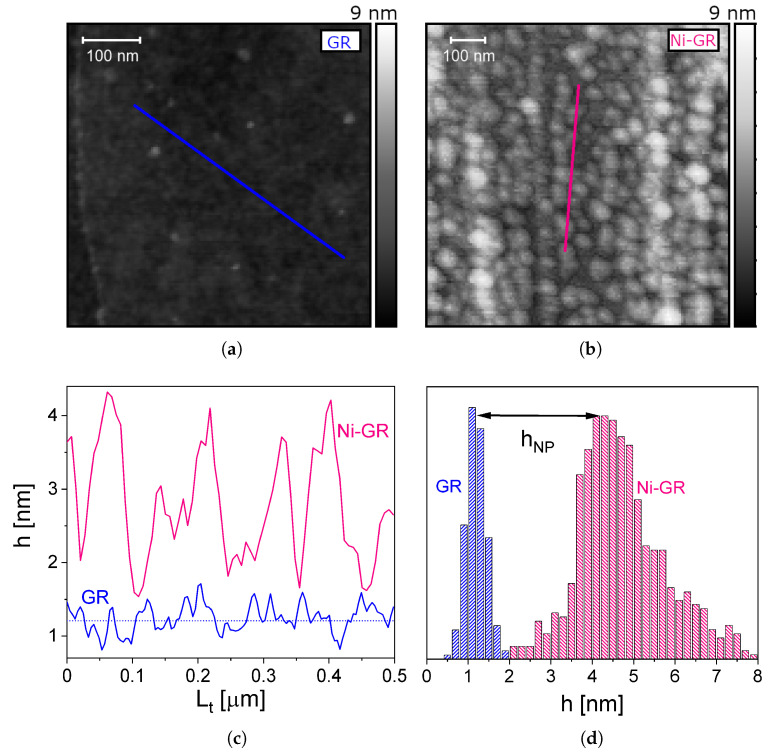
AFM images of the (**a**) graphene and (**b**) Ni-nanoparticle-decorated graphene samples with transversal cuts of the surface ($L_{t}$). (**c**) The heights of the transversal cut lines and (**d**) the distribution of heights of the graphene layer (blue) and of the Ni nanoparticles placed on top of graphene (red), as calculated by the watershed algorithm.

Atomic force microscopy (AFM) images are a high precision profile measurement of the height of the sample (the vertical position of the surface). Conversely, AFM shows in general a poor lateral resolution. There are three main factors that determine lateral precision: the range of interactions of the electrostatic potential, the cantilever tip radius, and the in-plane step size, which depends on the scan area [56,57,58,59]. In our case, the AFM tip with a diameter of 10 nm was unable to clearly resolve individual particles and its lateral information. However, we can use the very precise height measurements to estimate the geometrical characteristics of the nanoparticles. In Figure 1, we present AFM images, where the formation of the nanoparticles can be observed in Figure 1b compared to the graphene-only control sample in Figure 1a. From this image, using a watershed algorithm, we obtained the distribution of particle heights *h* shown in Figure 1d, where $h_{\mathrm{NP}}\approx 3\;nm$ is the average height of the nanoparticles. This means that, after annealing, the 2 Å thick layer of the thermally deposited nickel transforms into ∼3 nm tall nanoparticles. However, since the nanoparticles are randomly distributed over the graphene’s surface, it is necessary to study the possibility that they may form a percolative conductive channel. Within percolation theory [60,61,62], the formation of these channels is determined by the so-called percolation threshold, which is 50% in two dimensions. This means that, in order to form one or more conductive channels, it is necessary to have at least half of the area covered by metallic clusters. If we assume that the typical lateral size and density per unit area of the nanoparticles are given by *D* and λ, respectively, and by considering that the total volume of the nanoparticles is equivalent to the volume of the deposited layer of thickness *t*, we can estimate the area covered by the Ni nanoparticles as

(5) $$\begin{array}{c} f∼D^{2}\lambda ∼\frac{t}{h_{\mathrm{NP}}} \end{array}$$

Indeed, the precise values of the area coverage fraction are $f=t/h_{\mathrm{NP}}∼6.7$% for cubic particles, $f=3t/h_{\mathrm{NP}}∼20$% for pyramidal particles, and $f=3t/2h_{\mathrm{NP}}∼10$% for hemispherical nanoparticles. Therefore, even though we do not have precise information about the lateral geometry of the nanoparticles, we conclude that in any case the area coverage fraction is well below the percolation threshold of 50%. Therefore, no conductive channels are formed across the nanoparticles and the current is guaranteed to flow through the graphene ribbon. In the case of the more reasonable model of hemispherical nanoparticles, the typical lateral size would be $D=2h_{\mathrm{NP}}∼6\;nm$ and the area density $\lambda ∼3.5\times 10^{-3}\;nm^{-2}$. This implies that for hemispherical nanoparticles, the gap distance between the nanoparticles could be estimated as $d_{\mathrm{edge}}=\lambda^{-1/2}-D∼10\;nm$, thus explaining the inability to resolve individual nanoparticles in the AFM images.

**Figure 2:**
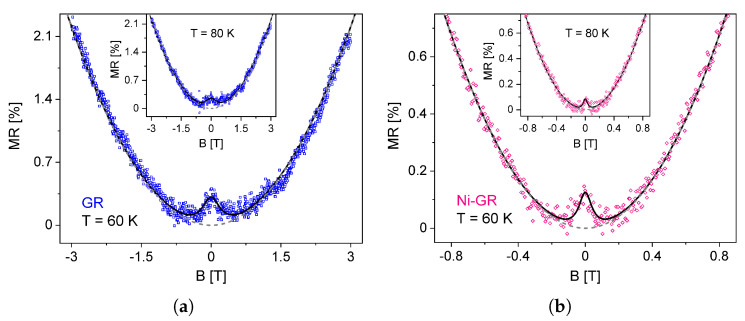
Magnetoresistance curves of (**a**) graphene and (**b**) Ni−nanoparticle−decorated graphene at temperatures of 60K and 80K (insets). Black solid lines are fittings with Equation (6) and dashed gray lines are the parabolic contribution from $\rho_{xx}\left(B\right)$.

Figure 2 shows the magnetoresistance (MR) of the GR and Ni-GR samples at temperatures of 60K and 80K. In these curves, it is possible to observe two field-dependent contributions: a well-defined parabolic behavior that can be found at high fields (typically above 0.5 T) and a narrow peak that appears superimposed at small fields. In order to disentangle both effects, we define the magnetoresistance as

(6) $$\begin{array}{cc} \mathrm{MR}(B) & =\frac{\rho \left(B\right)-\rho_{xx}\left(0\right)}{\rho_{xx}\left(0\right)}\\ \; & =\frac{\rho_{xx}\left(B\right)-\rho_{xx}\left(0\right)}{\rho_{xx}\left(0\right)}+\frac{\rho_{\mathrm{WL}}\left(B\right)}{\rho_{xx}\left(0\right)} \end{array}$$

thus clearly showing the two contributions. On the one hand, the overshoot of the resistivity caused by WL at low fields is described by the second term, such that $\mathrm{MR}\left(0\right)=\rho_{\mathrm{WL}}^{0}/\rho_{xx}\left(0\right)$ is the value of the peak in the MR at zero field. On the other hand, at high fields WL effects vanish and the parabolic MR is determined by the first term, associated with the EEI in $\rho_{xx}\left(B\right)$. Along with the MR data, in Figure 2, we show the successful fittings using the model described by Equation (6) using four relevant parameters, namely $\rho_{\mathrm{WL}}^{0}$, γ, $\rho_{xx}\left(0\right)$, and the curvature of the longitudinal resistivity, $C=\rho_{D}^{2}\mu^{2}\Delta \sigma_{\mathrm{EEI}}$.

### 4.1. Low Fields: Weak Localization

**Figure 3:**
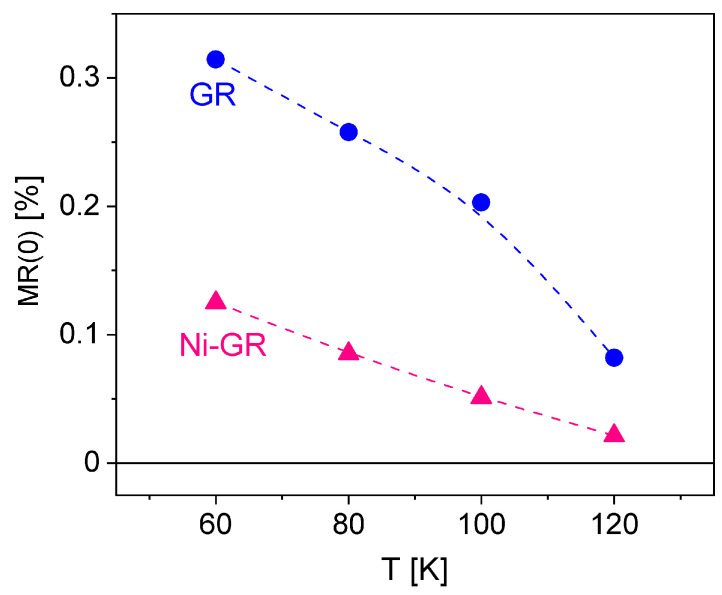
Zero field magnetoresistance vs. temperature for graphene (**blue**) and Ni-nanoparticle-decorated graphene (**red**).

There are two important features to observe in Figure 2. First, the magnitude of the WL peak decreases with increasing temperature. Second, it is clear that WL effects are stronger for the GR sample. From the fittings of the MR data, we were able to obtain the values of $\mathrm{MR}\left(0\right)=\rho_{\mathrm{WL}}^{0}/\rho_{xx}\left(0\right)$ as a function of temperature for both samples, as shown in Figure 3.

As the temperature increases, WL effects tend to vanish at temperatures larger than 130K. Since weak localization is a quantum phenomenon, increasing the temperature weakens phase coherence, thus naturally reducing the probability of backscattering. Indeed, previous works on exfoliated graphene have shown that quantum interference effects can survive up to temperatures as high as 200 K [63], in agreement with the present results. On the other hand, it is more interesting to note that the addition of the Ni nanoparticles on top of graphene greatly reduces WL effects by a factor of ∼3 as compared to the control graphene ribbon. It is well known that the presence of magnetic impurities in graphene has a strong impact on the ability of the electrons to maintain phase coherence. Such a reduction in WL in the Ni-GR sample then implies that there is a magnetic coupling between the graphene layer and the magnetic NPs, increasing the spin–orbit interaction [54] and weakening phase coherence.

### 4.2. High Fields: Electron–Electron Interaction

**Figure 4:**
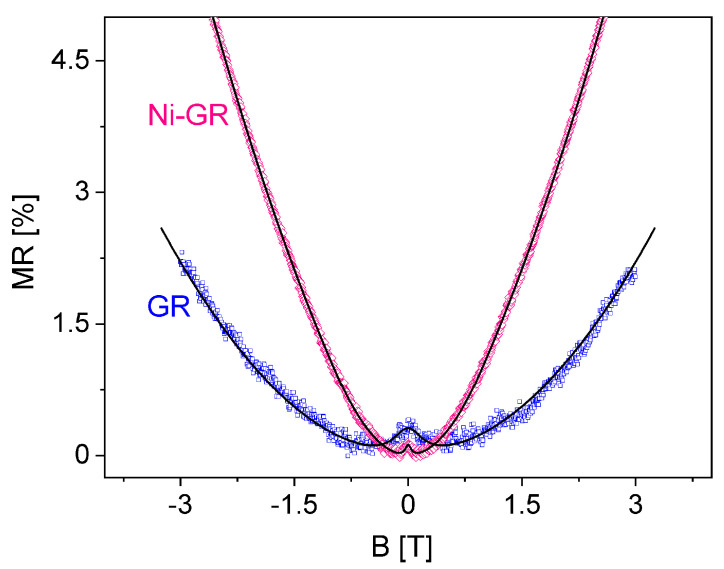
Magnetoresistance curves at high magnetic fields for graphene (**blue**) and Ni−nanoparticle−decorated graphene (**red**) at T=60K. The black solid lines represent the corresponding fittings with a purely quadratic behavior (GR) and with the added contribution of the interaction field $B_{i}$ (Ni−GR, see text).

In order to discuss the effect of the Ni NPs at high fields, in Figure 4, we present the MR curves of both samples at 60 K, where a much more important magnetic field dependence can be clearly distinguished for the Ni-nanoparticle-decorated graphene.

Nickel is a ferromagnetic material with a large Curie temperature in excess of 500K [64,65]. Small Ni nanoparticles present a superparamagnetic behavior with a small magnetic anisotropy, that allows to reach the saturation of the magnetic moment at applied magnetic fields of ∼0.5−1 T [64,65,66]. Therefore, we understand the enhanced high-field MR in the Ni-GR sample on the basis of a magnetic proximity effect of Ni on the graphene layer [67]. The magnetic moment of the Ni nanoparticles saturates in the same direction of the applied field; thus, under this proximity effect interpretation, the Ni–C magnetic coupling should manifest as an effective interaction field ($B_{i}$) added to the external field. Indeed, we were unable to fit the MR curve of the Ni-GR sample using the purely quadratic dependence of Equation (1). Instead, for the successful fitting of this sample, shown in Figure 4, we introduced the modification $B\to B+B_{i}$. We note that, on the contrary, the fitting for the GR sample was performed using the purely quadratic dependence of $\rho_{xx}\left(B\right)$ with no modifications.

From the fitting of the MR data of the Ni-GR sample, shown in Figure 4, we obtained $B_{i}\approx 2.1$ T. However, this is an effective field averaged over the surface of the graphene layer. Therefore, since the Ni nanoparticles cover only 10–15% of the graphene surface, the measure of the direct Ni–graphene exchange coupling is given by the effective (local) exchange field, $B_{\mathrm{ex}}=B_{i}/f∼15-20$ T. This is a large but very reasonable value for direct exchange interactions, and corresponds to a typical exchange splitting $\Delta \approx 2\mu_{B}B_{\mathrm{ex}}∼2\;me\;V$ and an exchange coupling constant $J∼4\mu_{B}^{2}B_{\mathrm{ex}}/M_{\mathrm{Ni}}$ of around 6meV ($M_{\mathrm{Ni}}∼0.6-0.7\;\mu_{B}$ is the atomic magnetic moment of Ni [68,69,70,71]). Similar proximity-induced exchange interactions of comparable magnitudes have also been observed in graphene covered with magnetic insulators. For example, for graphene layers coupled to EuO or CrCl${}_{3}$, exchange splittings of around 5 meV have been estimated [31,35]. Furthermore, for graphene/EuS interfaces, a large exchange field in excess of 14 T has been found [32], in agreement with our results. In our case, the large exchange constant, indicative of a strong orbital overlap between the 3d electrons of Ni and the π electrons of graphene [72], must be also responsible for the notable reduction in WL effects, as described previously.

## 5. Discussion

Our results show two effects that are directly associated with the magnetic coupling of the graphene with the Ni nanoparticles, i.e., the appearance of a large effective exchange field and the suppression of WL phenomena. However, the question remains open whether the intrinsic electronic properties of the underlying graphene were also modified or if the Ni–C interaction is only associated with a magnetic proximity effect. We hereby study and compare the electronic transport parameters of both the GR and Ni-GR samples. From the fittings of the MR data, we were able to obtain the curvature of the longitudinal resistivity, $C=\rho_{D}^{2}\mu^{2}\Delta \sigma_{\mathrm{EEI}}$ (see Equation (1)). This quantity is mainly determined by the mobility and scattering time of the conduction electrons, such that

(7) $$\begin{array}{c} \frac{C}{\rho_{xx}^{2}\left(0\right)}∼\mu^{2}\Delta \sigma_{EEI} \end{array}$$

**Figure 5:**
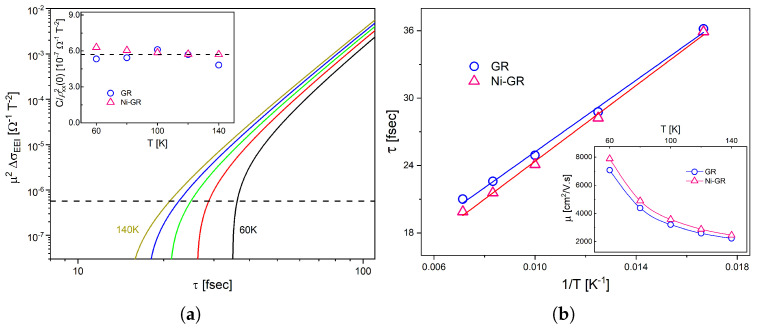
(**a**) Solid lines show the function $\mu^{2}\Delta \sigma_{\mathrm{EEI}}$ as a function of the scattering time at different temperatures between 60K and 140K. Inset: experimental results of $C/\rho_{xx}^{2}\left(0\right)$ as a function of temperature, as obtained from the fitting of the magnetoresistance data with Equation (6) (blue circles for graphene and red triangles for the Ni−nanoparticle−decorated graphene). The average value is $5.7\times 10^{-7}\;\Omega^{-1}T^{-2}$, represented in the main panel by the dashed line. (**b**) Scattering time of graphene and Ni−nanoparticle−decorated graphene as a function of inverse temperature. Inset: electron mobility as a function of temperature.

Notably, even though the MR data of the samples are very different and the fittings were carried out using two distinct models (with and without an exchange field), the curvatures resulted to be remarkably similar and essentially independent of temperature in the range 60–140 K. The inset of Figure 5a presents the experimental results, and both samples are within ∼10% of each other, with an average of $C/\rho_{xx}^{2}\left(0\right)∼5.7\times 10^{-7}\;\Omega^{-1}T^{-2}$. From these data we can estimate the scattering time and electron mobility of our graphene sheets.

In Equation (1) both the mobility and the electron–electron interaction correction depend on the scattering time and temperature. Using the usual Drude transport equations for resistivity and mobility, $\rho_{D}^{-1}=ne\mu$ and $\mu =ev_{F}\tau /\hbar \sqrt{\pi n}$, the electron mobility can be written as

(8) $$\begin{array}{c} \mu \left(T,\tau \right)=\frac{e^{3}v_{F}^{2}\rho_{D}\left(T\right)\tau^{2}}{\pi \hbar^{2}} \end{array}$$

where $v_{F}\approx 10^{6}\;m/s$ is the Fermi velocity. Therefore, by combining Equations (2) and (8), we calculated the curves of $\mu^{2}\Delta \sigma_{EEI}$, the right hand side of Equation (7), as a function of the scattering time τ at different temperatures between 60K and 140K, as shown in Figure 5a for the GR sample (the curves for the Ni-GR sample are very similar). From the intersection of theses curves with the experimental value of $C/\rho_{xx}^{2}\left(0\right)$, we obtained the scattering time as a function of temperature.

Figure 5b presents the results for the scattering time, which shows the typical 1/T dependence, while the inset of the same figure shows the calculated electron mobility. In both cases, the results demonstrate that the microscopic transport parameters are the same for both samples, i.e., they remain unchanged when the graphene layer is decorated with Ni nanoparticles. These transport parameters, with τ∼30 fs and $\mu ∼5000\;cm^{2}/Vs$ at intermediate temperatures, are consistent with the presence of moderate quantities of scattering centers in graphene [73,74]. In fact, the values of the scattering time shown in Figure 5b are an order of magnitude smaller than the typical dephasing time in graphene in the same temperature range [63]. This is indeed the optimal condition for the observation of WL effects at low magnetic fields, such as the ones shown in this work.

## 6. Conclusions

The magnetoresistance of graphene has a quadratic dependence with the magnetic field due to electron–electron interactions, and a zero-field peak due to a weak localization quantum effect. In this paper, we have studied the effect of ferromagnetic nickel nanoparticles on the resistivity of graphene. We show that the Ni nanoparticles produce a large magnetic exchange coupling with the underlying graphene layer. At low applied magnetic fields, the local exchange fields of the Ni nanoparticles likely introduce magnetic scattering centers, boosting the spin–orbit interaction and thus increasing the dephasing rate of the conduction electrons. The consequence is a reduction of the WL peak by a factor 3. Furthermore, at high magnetic fields the exchange coupling with the ferromagnetic nanoparticles introduces an effective field in graphene, which adds to the applied field and produces a very large MR response. However, the curvature of the parabolic magnetoresistance, controlled by the mobility and scattering time, remains the same for both the decorated and the non-decorated samples and agrees well with the expected values for graphene. From the MR curves, we obtained a Ni–graphene magnetic exchange constant of around 6 meV, in excellent agreement with previous estimations.

This work demonstrates the tunability of the magnetotransport properties of graphene via a strong coupling with ferromagnetic Ni nanoparticles. Therefore, our results show the potential of graphene-based magnetic heterostructures in the design of spintronic devices.

## Data Availability

All data generated or analyzed during this study are included in this published article.
